# Long-term clinical prognosis of 335 infant single-gene positive FEVR cases

**DOI:** 10.1186/s12886-022-02522-8

**Published:** 2022-08-02

**Authors:** Chunli Chen, Yizhe Cheng, Zhihan Zhang, Xiang Zhang, Jiakai Li, Peiquan Zhao, Xiaoyan Peng

**Affiliations:** 1 Beijing Tongren Eye Center, Beijing Tongren Hospital, Capital Medical University, Beijing, China; 2 Beijing Ophthalmology and Visual Science Key Laboratory, Beijing, China; 3 Beijing Institute of Ophthalmology, Beijing, China; 4 Department of Ophthalmology, Xinhua Hospital, Medicine School of Shanghai Jiaotong University, No. 1665, Kongjiang Road, Shanghai, 200092 People’s Republic of China; 5 Beijing Ophthalmology and Visual Science Key Laboratory, Beijing Tongren Eye Center, Beijing Tongren Hospital, Capital Medical University, No.1, Dongjiaominxiang Street, Dongcheng District, Beijing, 100005 People’s Republic of China

**Keywords:** FEVR, Gene, Clinical features, Relation between phenotypes and genotypes, Prognosis

## Abstract

**Purpose:**

To describe and analyze the clinical prognosis of infants diagnosed of familial exudative vitreoretinopathy (FEVR) with single gene mutation in long-term follow-up.

**Methods:**

A retrospective case study was conducted on 355 FEVR infants with single positive gene.

**Result:**

Of the 335 single-gene positive infant FEVR cases (under 3 years old), 20% (*n* = 67) was diagnosed of strabismus at first visit. Staging of various genotypes was different (*P* < 0.001). Patients with NDP mutations presented the most severe clinical phenotypes and patients with ZNF408 mutations presented the mildest clinical phenotypes. Most infants underwent surgery under 1 year old (5^th^ stage 75 of 108 [69.44%]). The axial length of different genotypes showed no significant difference (*P* = 0.2891). The 1^st^ to 3^rd^ stage cases were given intravitreal injection and/or retina photocoagulation with the last follow-up vision above 20/67. The 4^th^ to 5^th^ stage cases received the transcorneal vitrectomy with lensectomy or lens sparing vitrectomy (LSV), whose lens maintained transparent after LSV (11/14[78.58%]). After 2 to 10 years of follow-up, 37.96% (41/108) of post-surgery cases showed retinal funnel-like unfold and posterior pole unfold, 69.57% (16/ 23) of which received second surgery for closure of pupil with good prognosis. At the last follow-up, 20% (60/300) were with vision above 20/200.

**Conclusion:**

LRP5 gene mutation was the most common mutation in FEVR patients. The severity of the clinical phenotype varied with different gene mutations. The main surgical methods for cases at Stage 4–5 were transcorneal vitrectomy with lensectomy or LSV. The earlier FEVR occurred, the worse prognosis would be. Active surgical intervention and lens sparing were necessary for cases at Stage 4–5.

## Introduction

Familial Exudative Vitreoretinopathy (FEVR) is a rare hereditary eye disease, with high prevalence of 13 ~ 16% in children blind-causing eye diseases [1], first described by Criswick and Schepens in 1969 [2]. According to the manifestation of retinal abnormalities, FEVR is classified into 5 stages, which was first proposed by Pendergast [3]. FEVR is predominantly inherited in an autosomal dominant manner, as well as in autosomal recessive and X-linked recessive manner. At present, 6 acknowledged genes are responsible for the disorder, including FZD4, NDP, LRP5, TSPAN12, ZNF408 and KIF11. Typically, FEVR is regarded as a bilateral disease manifesting all the patterns of the Mendelian inheritance. There is also a certain proportion of unilateral FEVR, ranging from 3.22%-15% [4]. The underlying mechanisms of unilateral onset is elusive, possibly being related to epigenetics. The typical clinical features of FEVR are peripheral avascular retina, especially peripheral V-shaped avascular retina on the temporal side [5]. Retinal detachment (RD) is the most common complication of FEVR with a proportion of 21 ~ 64% [6]. The progressive tractional retinal detachment will finally develop into retinal fold, also called ‘falciform fold’, which is a typical manifestation of severe stage. The clinical features of FEVR include diverse characteristics, incomplete penetrance and asymmetry of disease [7]. Between probands and their other family members, there exists different severity of FEVR [8]. Combining previous studies with our massive clinical data, we have found that FEVR presents diverse clinical manifestations, pathological processes and genetic patterns. Therefore, we conduct a study to summarize relation between the clinical manifestations and genotypes in patients under 3 years old during long-term observation.

## Methods

### Study design

This study was conducted as a retrospective cases study in which the clinical manifestations of infant FEVR cases with different genotypes in a long-term follow-up was described and analyzed. This study critically complied with the Declaration of Helsinki and the guidelines for specimen collection of human genetic diseases promulgated by the Ministry of Health of China. All guardians of minors signed an informed consent form.

### Participants

Infant participants (*N*= 355, under 3 years old) with a diagnosis of FEVR and single positive gene were recruited from the Department of Ophthalmology, Xinhua Hospital, Shanghai Jiaotong University and Department of Ophthalmology, Beijing Tongren Hospital, Capital Medical University between January 2010 and December 2018. The inclusion criteria were as follows. All the time of presentation of the patients were under 3 years old. Patients showed clinical features of FEVR that are confirmed by ultra-wide fundus fluorescein angiography, and had likely pathogenic or pathogenic mutations detected by genetic testing among 6 acknowledged pathogenic genes of FEVR (LRP5, FZD4, ZNF408, NDP, TSPAN12 and KIF11). The baseline data were collected, including chief complaints, sex, laterality, visual acuity, intraocular pressure, ophthalmic examination, family history, birth history, treatment history and so on. Other diseases that have similar clinical features with FEVR were excluded. Based on clinical features, imaging manifestations and gene testing results, the diagnosis was made by experienced doctors. All the patients were classified into 5 stages, including Stage 0–5, according to their clinical manifestations and severity. Our classification is based on the Pendergast’s classification [3]. Pendergast’s classification defined Stage 1 as ‘avascular retinal periphery without extraretinal vascularization’, Stage 2 as ‘avascular retinal periphery with extraretinal vascularization’, Stage 3 as ‘subtotal retinal detachment not involving fovea’, Stage 4 as ‘subtotal retinal detachment involving fovea’ and Stage 5 as ‘total retinal detachment’. Further, based on the severity of manifestation, we defined stages 1–3 as mild types and stage 4–5 as severe types [9]. The normal eyes were regarded as Stage 0. For better description and understanding, the patients were subdivided into several groups, including patients with monocular involvement (MI), of which fellow eyes have no areas of avascularity strictly based on the fundus fluorescence angiography (FFA), patients with binocular involvement and mild manifestation (Stage 1–3) in both sides (BM), patients with binocular involvement and severe (Stage 4–5) manifestation in both sides (BS), and patients with binocular involvement and mild manifestation in one eye and severe manifestation in the other eye (B[M + S]). All DNA samples were extracted from peripheral whole-blood samples (2 mL) collected from the participants, then the enrichment of the DNA fragments related to FEVR was performed on the DNA sales by using targeted-region capture. Then, targeted next-generation sequencing (NGS; MyGenetics, China Beijing) was performed. Using Sanger sequencing, the samples of parents or other family members were validated. All the genetic data were analyzed to identify reported variants and estimate the pathogenicity. Family segregation was also assessed.

### Interventions

All surgeries were performed by experienced surgeons. According to patients’ condition, different surgical methods were performed, including closed vitrectomy (Closed-V) with lensectomy, pars plana vitrectomy (PPV) combined with silicone oil or C3F8 injection, lens-sparing vitrectomy (LSV), scleral buckling (SB), intravitreal injection of ranibizumab (IVR) and strabotomy.

- (1) Closed-V is a surgery procedure characterized by a limbus approach. The procedures included lensectomy combined with vitrectomy, and staged lensectomy and vitrectomy. Lensectomy is performed if the anterior chamber is shallow or flat with corneal edema and secondary glaucoma is complicated, or the lens is opaque and vitrectomy is performed if the fundus is visible and the conditions of fundus is stable. For lensectomy, a 20-gauge cannula was inserted to keep continuous infusion at the temporal limbus through the iris root instead of the pars plana to avoid damage to the retina. However, if the fundus is invisible because of corneal opacity or if preretinal vascular fibrovascular proliferation is in the active phase, which could cause severe vitreous hemorrhage, a second vitrectomy procedure is performed if corneal edema and corneal opacity are relieved several weeks after lensectomy during the follow-up period, which is called staged lensectomy and vitrectomy [10, 11].
- (2) LSV consisted of standard 23-gauge three-port pars plana/plicata (incomplete development of the pars plana in infants) lens-sparing vitrectomy. In brief, after conjunctival peritomy, sclerotomies were performed 0.5 to 1 mm away from the limbus. To avoid lens damage, the direction of the sclerotomy was more vertical rather than toward the center of the eyeball. LSV is applicable for patients with a transparent lens and complete posterior vitreous detachment could not be performed for patients with only local fibrovascular proliferation [10–13].
- (3) SB is applicable for patients without severe complications (corneal edema, corneal degeneration and cataract), such as local tractional retinal detachment (Stage 3) or rhegmatogenous retinal detachment.
- (4) IVR is an adjuvant but significant treatment in surgical interventions and works by inhibiting vasoactivity. IVR 1 week before operation can inhibit the neovascularization and avoid the formation of retinal fibrovascular proliferation (one of complications of IVR), allowing the surgeon to perform the procedure in a relatively inactive or "quiet" eye. IVR is performed for patients with neovascularization at Stage 2–4, while subretinal injection of anti-VEGF could be performed for active retinal hemorrhage at Stage 5 [14, 15]. Laser coagulation and transscleral cryotherapy were also performed if appropriate. Laser coagulation, transscleral cryotherapy and IVR were not double-counted when these procedures were used as additional and necessary preoperative or intraoperative treatment.
- (5) The patients that had strabotomy, had the surgery done elsewhere and the retinal abnormalities were identified upon referral to our center.
- (6) PPV was performed to remove the epiretinal traction with complete posterior vitreous detachment, followed by silicone oil or C3F8 injection.

Laser coagulation and transscleral cryotherapy were also performed if appropriate. Laser coagulation, transscleral cryotherapy and IVR were not double-counted when these procedures were used as additional and necessary preoperative or intraoperative treatment. Routine anti-inflammatory and anti-infective treatments are performed after the operation. After the surgery, vision acuity, intraocular pressure, anterior segment, fundus and complications were first to observe, and detailed follow-up visits were recorded.

### Statistical analysis

All statistical analyses were performed using SPSS (Statistical Product and Service Solutions, Version 25.0; IBM Corporation, Chicago, IL, USA). The normally distributed measurement data was presented as (mean ± standard deviation), non-normally distributed measurement data was presented as median, minimum and maximum and the count data was presented bas percentage (%). The Kruskal–Wallis tests were used to investigate the correlation between gene mutations and clinical features. The χ2 tests were used for comparing the difference of gene mutations between FEVR patients and normal population and t tests for comparison of groups. The two-way ANOVA test to exclude the effect of clinical stage on the results. *P* < 0.05 was considered statistically significant.

## Results

### Clinical statistics of FEVR patients

**Fig. 1:**
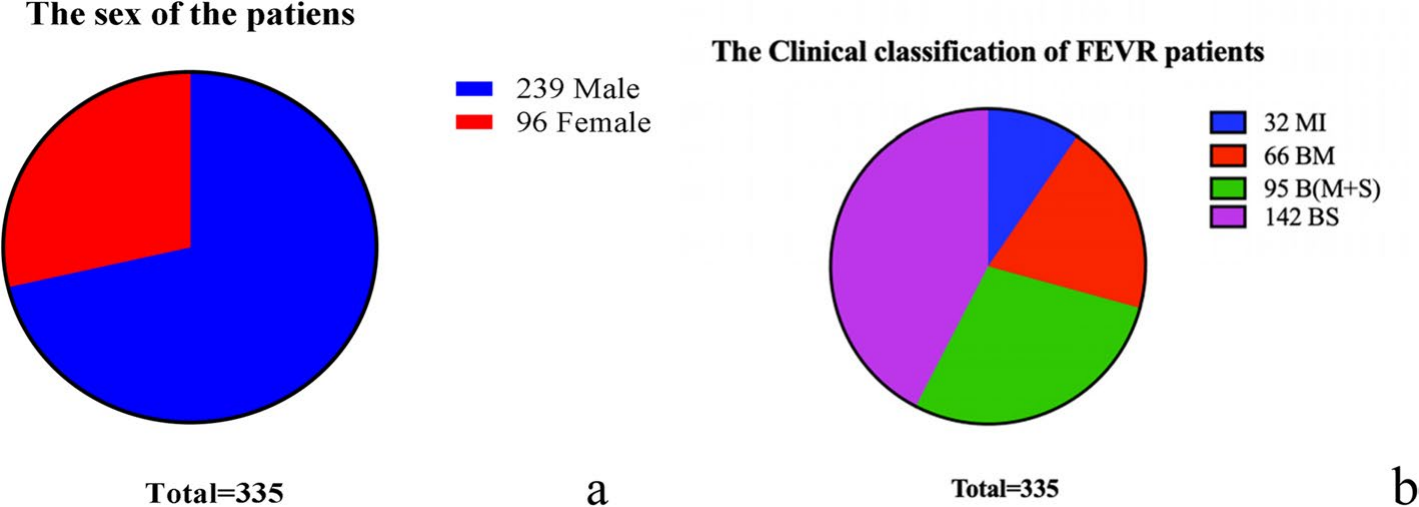
Clinical statistics of FEVR patients. Patients with monocular involvement (MI), patients with binocular involvement and mild manifestation (Stage 1–3) in both sides (BM), patients with binocular involvement and severe (Stage 4–5) manifestation in both sides (BS), and patients with binocular involvement and mild manifestation in one eye and severe manifestation in the other eye (B[M + S])

There are 239 males and 96 females, with a male-to-female ratio of 2.48:1 (the male-to-female ratio was 1.99 :1 after eliminating 48 cases with NDP mutation) in 335 single gene positive FEVR infants (Figure 1a). All the patients were subdivided into 4 groups, including 32 patients (9.6%) with monocular involvement (MI), 66 patients with binocular involvement and mild manifestation (BM), 95 patients with binocular involvement and mild manifestation in one eye and severe manifestation in the other eye [B(M+S)] and 142 patients with binocular involvement and severe manifestation (BS). Therefore, in our study, patients with severe bilateral FEVR accounted for the majority, while patients with unilateral FEVR accounted for the minority (Figure 1b).

#### Correlation between clinical phenotype and mutation

**Fig. 2:**
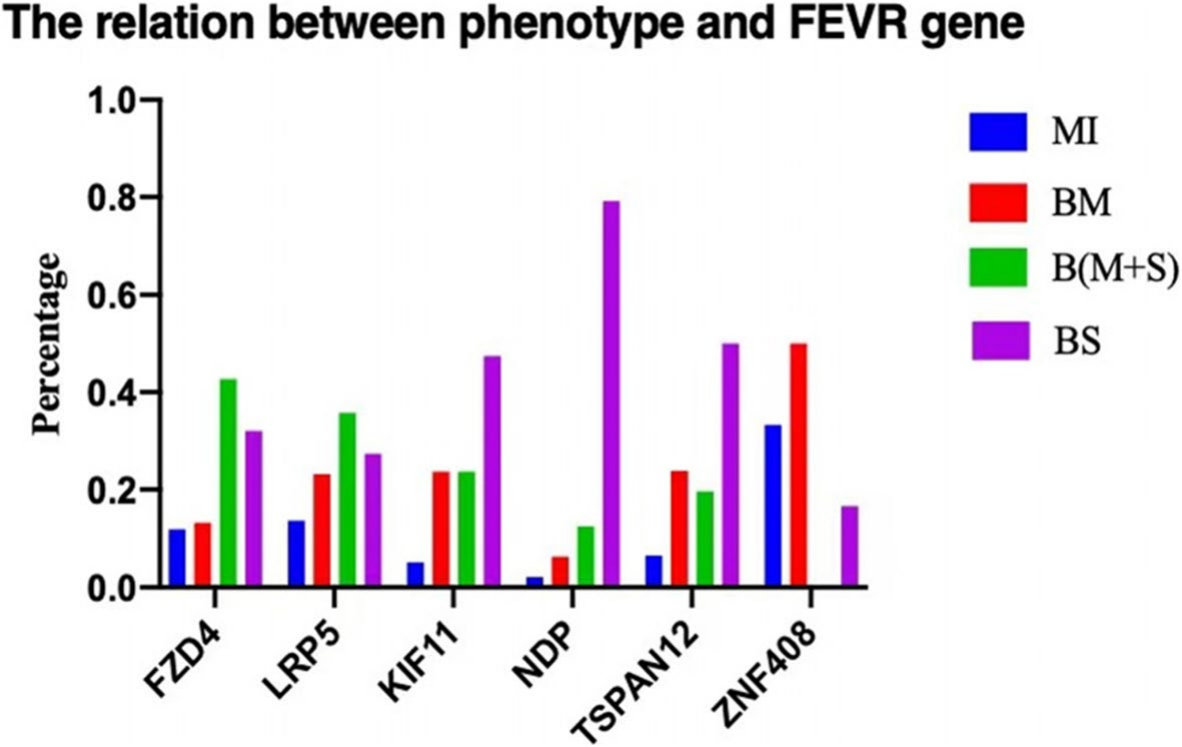
The correlation between clinical phenotype and mutant genes. Patients with monocular involvement (MI), patients with binocular involvement and mild manifestation (Stage 1–3) in both sides (BM), patients with binocular involvement and severe (Stage 4–5) manifestation in both sides (BS), and patients with binocular involvement and mild manifestation in one eye and severe manifestation in the other eye [B(M + S)]

Probands with mutations of LRP5, FZD4, NDP, TSPAN12 and ZNF408 accounted for 28.3% (95 of 335), 22.4% (75 of 335), 14.3% (48 of 335), 13.7% (46 of 335) and 3.58% (12 of 335). LRP5 gene mutation was the most common mutation in FEVR patients. The severity of the clinical phenotype varied with different gene mutations. Patients with NDP mutations presented the most severe clinical phenotypes, and 38 of 48(79.2%) belonged to BS group. On the contrary, patients with ZNF408 mutations presented the mildest clinical phenotypes, and 6 of 12 (50%) belonged to BM group. Spontaneous mutation of KIF11 took over the majority, accompanied by microcephaly and choroidal atrophy. TSPAN12 had the highest familial hereditary ratio (Fig. 2, Table 1).

**Table 1:** Clinical stages and severity in different single gene mutations

Gene	Stage zero		Stage one		Stage two		Stage three		Stage four		Stage five	
	Eyes	Percentage	Eyes	Percentage	Eyes	Percentage	Eyes	Percentage	Eyes	Percentage	Eyes	Percentage
LRP5	12	6.3%	40	21.1%	23	12.1%	16	8.4%	61	32.1%	38	20.0%
FZD4	9	6.0%	28	18.7%	17	11.3%	8	5.3%	57	38.0%	31	20.7%
KIF11	3	2.5%	9	7.6%	27	22.9%	4	3.4%	50	42.4%	25	21.2%
NDP	1	1.0%	3	3.1%	9	9.4%	1	1.0%	17	17.8%	65	67.7%
TSPAN12	3	3.3%	7	7.6%	23	25.0%	2	2.2%	51	55.4%	6	6.5%
ZNF408	4	16.7%	1	4.2%	9	37.5%	2	8.3%	5	20.8%	3	12.5%

#### Clinical prognosis in diverse genotypes

##### The characteristic of clinical stages in different single gene mutations

In MI group, 12 out of 95 patients in MI group having LRP5 mutation, patients with ZNF408 had the highest proportion (33.3, 4/12). The NDP mutation had the highest proportion of bilateral involvement. Staging of different genotypes was different (*P* < 0.001). In NDP genotype, the proportion of severe stage was the highest with a meaningful statistical difference (*P* < 0.05). Normal stage in ZNF408 genotypes showed the highest proportion (16.7%), followed by LRP5(6.3%), with a meaningful statistical difference (*P* = 0.029) compared with KIF11 genotype and no significant difference compared with other genotypes (*P* > 0.05). χ2 tests showed a significant difference among stages in different genotypes, which means proportions of different stages in different genotypes were not completely consistent. (Table 1).

##### Axial length of 335 single-gene infant FEVR cases with different genotypes

**Fig. 3:**
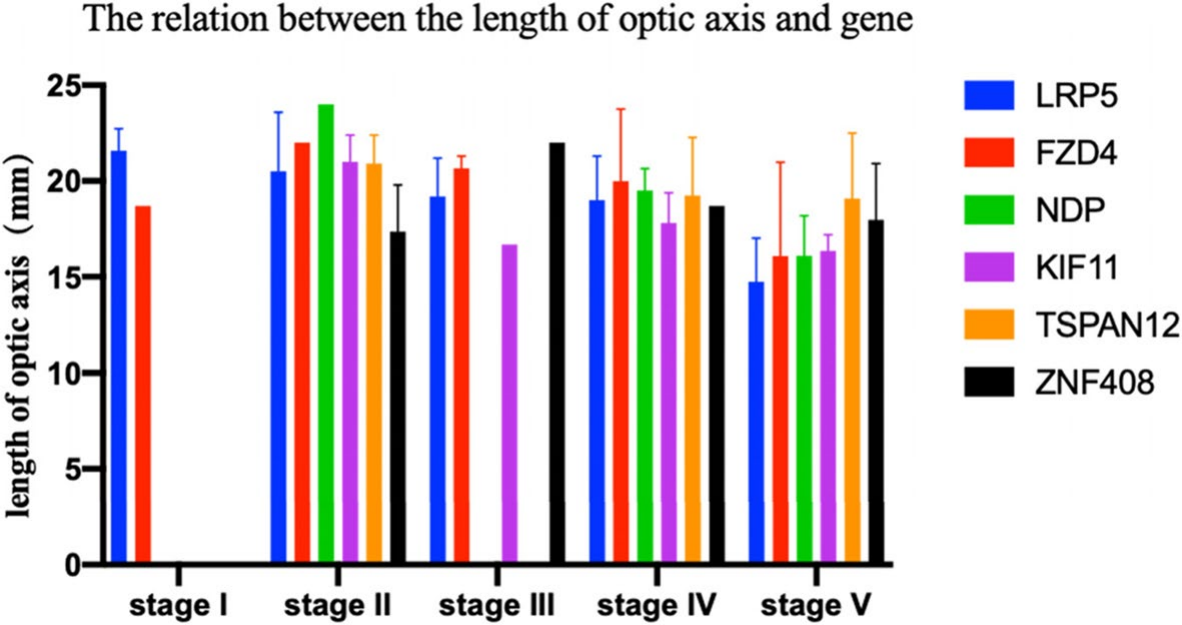
The relation between the length of optic axis and gene

In order to analyze the correlation between the gene mutation and the axial length of the eye, we chose the eye with shorter length for statistical analysis, speculating that the shorter ones are the eyes that is mostly involved. Due to different genotypes correspond to different distribution of clinical stages, we apply two-way ANOVA test to find whether the type of gene mutation independently influences the axial length of the eye. There was no significant difference in the axial length of different genotypes (*P* = 0.2891) (Fig. 3, Table 2). Instead, clinical stage shows a significant relation with axial length (*P* < 0.0001). Statistical results showed that there was no significant correlation between genotype and axial length in patients with the same clinical stage. The genotypes more likely exerted influence on clinical phenotypes by affecting the distribution of clinical stage.

**Table 2:** The axial length in patients with different genotypes and stages

	LRP5			FZD4			NDP			KIF11			TSPAN12			ZNF408		
Stage	Mean AL	SD	N	Mean AL	SD	N	Mean AL	SD	N	Mean AL	SD	N	Mean AL	SD	N	Mean AL	SD	N
I	21.58	1.15	4	18.70	0.00	1	-	-	-	-	-	-	-	-	-	-	-	-
II	20.49	3.10	8	22.00	0.00	1	24.00	0.00	2	21.00	1.40	8	20.90	1.50	5	17.35	2.45	2
III	19.19	2.00	10	20.65	0.65	2	-	-	-	16.70	0.00	1	-	-	-	22.00	0.00	2
IV	19.00	2.29	43	19.99	3.76	27	19.51	1.12	8	17.82	1.58	22	19.25	3.03	22	18.70	0.00	1
V	14.74	2.28	28	16.09	4.89	20	16.10	2.09	28	16.36	0.83	12	19.10	3.40	4	17.97	2.93	3

*AL* Axial length, *SD* Standard deviation

##### Treatment for single-gene FEVR infants with different genotypes and stages

The difference among patients with different genotypes at different stages failed to reach the significant statistical difference. Surgeries including closed vitrectomy (Closed-V) with lensectomy, pars plana vitrectomy (PPV) combined with silicone oil or C3F8 injection, lens sparing vitrectomy (LSV), scleral buckling (SB), intravitreal injection of ranibizumab (IVR), strabotomy, laser coagulation and cryotherapy were performed appropriately. The proportion of surgical treatment was much higher in patients under 1-year-old and 69.44% (75 of 108) of them were at Stage 5. 78.58% (11 of 14) of the lens of the affected eyes maintained transparent after the treatment of LSV. About 37.03% (40 of 108) of post-surgery patients showed prognosis of retinal funnel-like unfold and posterior pole unfold, which mostly received Closed-V. The earlier FEVR occurred, the worse the prognosis would be (Table 3).

**Table 3:** The treatment of FEVR

Gene	Closed-V		LSV		PPV + Silicone oil/C3F8		SB		Cryotherapy		IVR		Laser coagulation		Strabotomy	
	Eyes	Percentage	Eyes	Percentage	Eyes	Percentage	Eyes	Percentage	Eyes	Percentage	Eyes	Percentage	Eyes	Percentage	Eyes	Percentage
LRP5 (*n* = 190)	22	11.6%	6	3.2%	9	4.7%	2	1.1%	1	0.5%	15	7.9%	17	8.9%	1	0.5%
FZD4 (*n* = 150)	20	13.3%	3	2.0%	6	4.0%	0	0.0%	0	0.0%	3	2.0%	11	7.3%	0	0.0%
KIF11 (*n* = 118)	19	16.1%	0	0.0%	3	2.5%	2	1.7%	0	0.0%	3	2.5%	2	1.7%	2	1.7%
NDP (*n* = 96)	30	31.3%	4	4.2%	2	2.1%	0	0.0%	0	0.0%	14	14.6%	5	5.2%	0	0.0%
TSPAN12 (*n* = 92)	6	6.5%	1	1.1%	1	1.1%	0	0.0%	1	1.1%	6	6.5%	4	4.3%	1	1.1%
ZNF408 (*n* = 24)	4	16.7%	0	0.0%	1	4.2%	0	0.0%	0	0.0%	0	0.0%	2	8.3%	0	0.0%

*Closed-V* Closed vitrectomy, *LSV* Lens-sparing vitrectomy, *PPV* Pars plana vitrectomy, *SB* Scleral buckling, *IVR* Intravitreal injection of ranibizumab

##### Prognosis of 335 single-gene FEVR infant cases with different genotypes

**Fig. 4:**
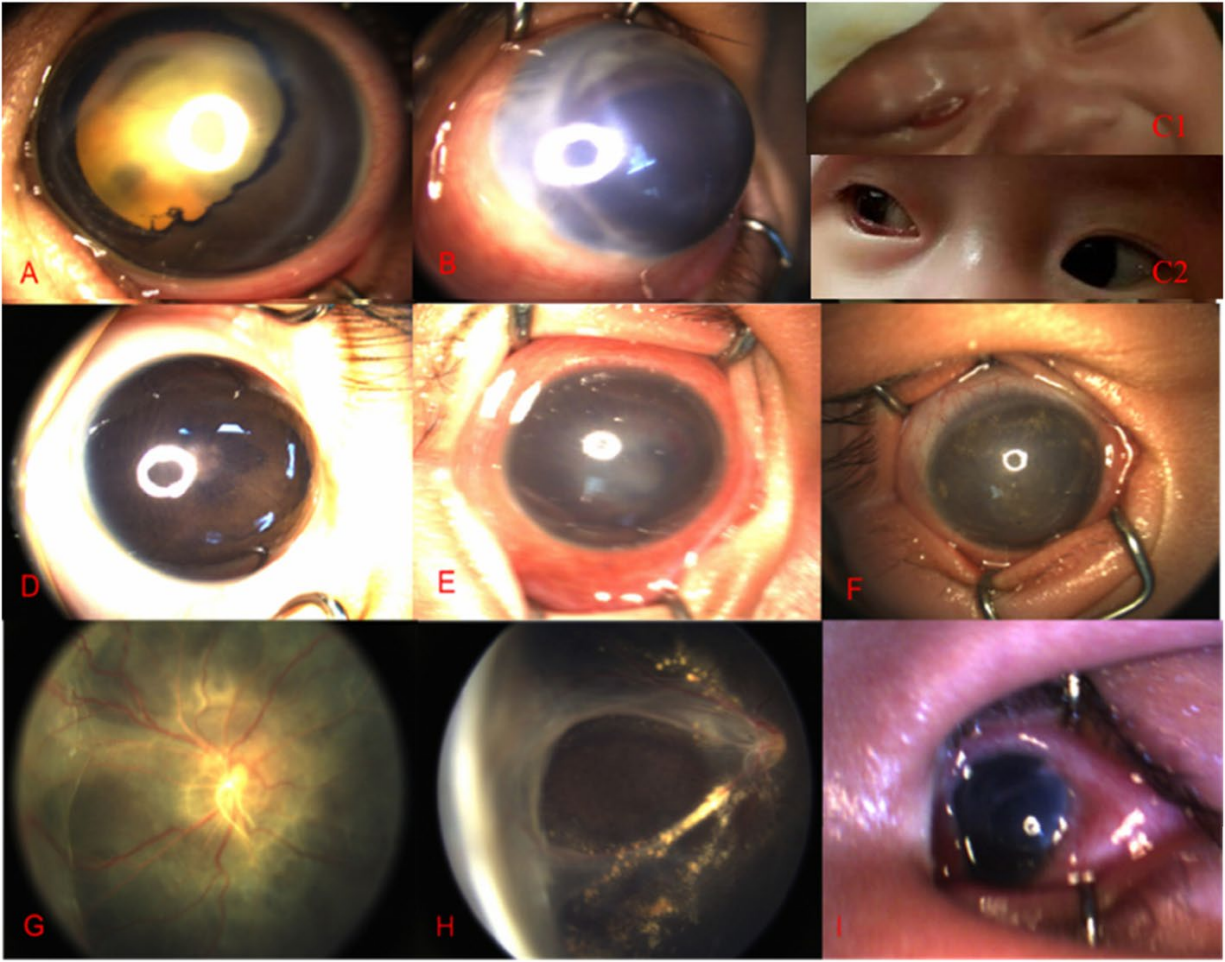
Picture A showed corneal degeneration and valgus collar of pupil. Picture B showed secondary glaucoma, becoming bigger and proptosis of the eyeball, mild corneal edema and disappearance of anterior chamber. Picture C1 showed obvious increase of IOP and orbital-celluitis-like change. Picture C2 showed smaller size of the right eye after high IOP for 3 days. Picture D showed closure of pupil and disappearance. Picture E showed corneal edema, anterior chamber disappearing with hemorrhage. Picture F showed plenty of cholesterol crystallization in the anterior chamber. Picture G showed flat posterior pole after surgery. Picture H showed funnel-shaped unfold after surgery. Picture I showed atrophy of the eyeball

The patients at Stage 1 visited biannually. The normal fellow eye (Stage 0) of the patients with monocular involvement and eyes at Stage 1 were not given any interventions in the follow-up period and remained in stable conditions (18.1%, 121 of 670). The patients at Stage 2-3 visited per 3 or 6 months, with an average follow-up time of 2-10 years. In these stable eyes, LRP5 mutation accounts for the highest percentage of 27.7% and NDP mutation accounts for the minority of 4.2%. Of total of 670 eyes, only 20.9% of affected eyes are mild types (Stage 2-3) and 32.8% of affected eyes were given interventions (laser coagulation, IVR and LSV). In follow-up, only 10% of infants evolved from Stage 2A to 2B, and then they were stabilized after laser treatment with no progression to Stage 4-5. However, there were 61% of patients with severe manifestation at presentation (Stage 4-5). At first visit, 56.2% of parents of infants with severe manifestation abandoned treatment because of the poor prognosis. By contrast, 37.96% of the rest infants receiving treatment showed resolution of funnel-like folding of retina involving posterior pole. The NDP mutations led to the worst prognosis (Table 4, Fig. 4). 37.96% (41 of 108) of post-surgery cases showed prognosis of retinal funnel-like unfold and posterior pole unfold, while could be accompanied by mild complications like closure of pupil and cornea dystrophy, severe complications like hyphema, secondary glaucoma, cholesterol crystallization, eyeball atrophy and so on. About 69.57% (16 of 23) of second surgery was performed because of pupil closure. At the last follow-up, 20% of infants at Stage 4 to 5 had vision above 20/200.

**Table 4:** The relation between prognosis and gene

Gene	Funnel-like unfold		Posterior pole unfold		Pupil closure		Secondary glaucoma		Corneal degeneration		Hyphema		Ocular atrophy	
	Eyes	Percentage	Eyes	Percentage	Eyes	Percentage	Eyes	Percentage	Eyes	Percentage	Eyes	Percentage	Eyes	Percentage
LRP5	8	4.2%	3	1.6%	4	2.1%	4	2.1%	3	1.6%	3	1.6%	5	2.6%
FZD4	5	3.3%	0	0.0%	8	5.3%	0	0.0%	2	1.3%	3	2.0%	0	0.0%
KIF11	10	8.5%	0	0.0%	1	0.8%	0	0.0%	0	0.0%	0	0.0%	0	0.0%
NDP	10	10.4%	0	0.0%	15	15.6%	0	0.0%	6	6.3%	3	3.1%	8	8.3%
TSPAN12	3	3.3%	0	0.0%	0	0.0%	1	1.1%	0	0.0%	0	0.0%	0	0.0%
ZNF408	2	8.3%	0	0.0%	2	8.3%	2	8.3%	0	0.0%	0	0.0%	0	0.0%

## Discussion

FEVR is a hereditary disorder involving abnormal retinal vascular development and has various clinical manifestations [16]. Up to now, it is acknowledged that 6 pathogenic genes are associated with FEVR, including LRP5 (Autosomal recessive or scattered inheritance gene), FZD4 and TSPAN12 (Autosomal dominant or recessive gene), ZNF408 (Autosomal dominant gene), NDP (X-Linked recessive gene) and KIF11 which was found recently and was related to the microcephaly-lymphedema-chorioretinal dysplasia (MLCRD) syndrome. It was reported that 40 ~ 50% of FEVR cases were related to the mentioned genes [17].

Our study probably enrolled the biggest cohort of FEVR infant patients by clinical collection and genetic-screening technology. Based on the evidence of gene chip sequencing analysis and genotype–phenotype co-separation [18, 19], 335 infant cases were detected as positive single-gene. About 78.8% of probands carried genes that is affiliated to the family of Wnt/Norrin signaling pathway. About 17.61% (59 of 335) of probands carried KIF11 gene and only 3.58% (12 of 335) of probands carried ZNF408. The results showed that FEVR induced by NDP and TSPAN12 mutations had a better symmetry than that induced by LRP5 and FZD4. Patients with NDP mutations showed severer clinical manifestations, followed by patients with TSPAN12 mutations. These findings were similar with findings of Tang et al. [20] Tang et al.found that FEVR patients with TSPAN12 mutations showed severe manifestation and rapid progression. The young patients showed severer clinical manifestation than that of adult patients. In our study, 58.1% of FEVR infant patients were over Stage 4. In addition, FEVR was a progressive disease. As the age of probands getting older, the severity of FEVR would accordingly change, which contributed to the different stages of FEVR. The higher clinical stages and the shorter axial length were, the severer clinical manifestation would be.

The treatment of FEVR included clinical observation, laser coagulation, intravitreal injection of anti-VEGF drugs and surgery [10, 12, 21–31]. Fundus fluorescein angiography (FFA) is a significant examination for helping diagnosis and deciding treatment. Clinical observation is suitable for patients without symptoms or leakage point on FFA. Previous studies suggested that only close follow up was required for patients over 3 years old and whose affected eye was at stage 2A or milder. However, argon laser coagulation is actively used for the eyes that are at stage 2B, accompanied by intraretinal and subretinal exudation or retinal neovascularization [25]. Laser coagulation is appropriate for the eyes with leakage on FFA, which can enclose the leaking blood vessel ends, reduce exudation and prevent mechanical traction after development of fibrous membrane. Intravitreal injection of anti-VEGF drugs is used for the eyes with broad exudation and neovascularization and the eyes that are hard to receive laser coagulation. Anti-VEGF drugs can reduce development of neovascularization and exudation, and suppress progression of FEVR. In our study, the cases at Stage 1 to 3 were mostly given intravitreal injection and/or retina photocoagulation with the last follow-up vision above 20/67. Surgery intervention is an important treatment for severe complications of progressive FEVR, such as vitreous hemorrhage, retinal tear or retinal detachment, secondary epiretinal membrane, secondary cataract or glaucoma and so on. In our study, appropriate surgery was chosen for different severity of FEVR patients, which was good for alleviating the complications of surgery. The patients under one year old accounted for the majority with a proportion of 69.44% for the patients at Stage 4 to 5, which demonstrated that the younger patients showed severer manifestation and close follow-up was necessary for patients under three years old. The stage 4^th^ to 5^th^patients were treated with the transcorneal vitrectomy with lensectomy or LSV, whose lens maintained transparent after LSV (11 of 14[78.58%]) corresponding to the previous study (74.1%) [11]. 37.03% of postoperative patients could achieve retinal unfold and partial retinal attachment after surgery. However, after long-term follow up and clinical observation, we comprehensively found that some patients might need to receive second surgery because of severe postoperative reaction, such as hemorrhage, exudation and fibrous membrane in pupil. 69.54% of them received second surgery owing to pupil closure. At the last follow-up, 20% (60 of 300) were with vision above 20/200 and under stable statement. Therefore, present study [10–13] recommended staged lensectomy and PPV are meaningful to alleviate the postoperative reaction and restore the depth of anterior chamber. For patients with occurrence of severe complications, we suggest that surgery is needed to maintain eyeball integrity, treat complications, prevent the occurrence of retinal detachment and maximize the restoration of visual function.

After analyzing the clinical features of 202 FEVR cases, Zhao et al. found that RD accounted for 33.6% in FEVR patients and 32.92% of affected eyes were misdiagnosed as simple RD. [32] The rate of correct diagnosis at first visit were 37.13% [27]. Our study dominantly focused on the infant FEVR patients that were under 3 years old. 20% (67 of 335) of infant cases were initially diagnosed as strabismus and ophthalmologists ignored the abnormalities of fundus. In addition to simple strabismus, a certain percentage of strabismus is caused by fundus diseases, such as persistent hyperplastic primary vitreous, X-linked retinoschisis, congenital macular hypoplasia, optic nerve hypoplasia, retinitis pigmentosa, incontinentia pigmenti achromians, Norrie disease, Coats’ disease and so on. These cases reminded ophthalmologists to examine patients’ fundus comprehensively when doctors meet the patients with a diagnosis of strabismus. If necessary, examining fundus of parents or gene testing is meaningful for avoiding misdiagnosis or late diagnosis resulting in severe FEVR. Early screening is an important way of early diagnosis for FEVR and treatment. If the progression of FEVR is stable and remain at Stage 1, the visual function can still be maintained. By contrast, if the progression of FEVR keep developing and causes damage to retina, the prognosis may not be optimistic. FEVR is a disease with extremely diverse manifestations, ranging from none of complaints or completely normal fundus to blindness and atrophy of the eyeball due to vitreous hemorrhage (VH) and RD. FEVR has a long time of development. The onset of FEVR happened when patients are in their infancy. The infant patients seldom showed obvious symptoms and manifestations in the early course of FEVR. At the late course, severe vision loss or even blindness happened due to VH or RD. Sometimes patients in the stationary phase could develop into active phase. Comprehensive fundus examination should be conducted on infants with strabismus. Long-time follow up or follow-up through life is consequently necessary for FEVR patients. The illness of some patients might intend to be active, even after they received treatments [33, 34]. Once the doctors perceive the advancement of the illness, patients should be given treatment immediately to avoid losing their visual function.

Considering the retrospective study, the limitations of our study are as follows. First, although our study enrolled a relatively large sample size, FEVR is a rare disease, and more patients probably provided strongly evidence-based clues. Second, due to FEVR is a disease that has a long development through life, the patients under 3 years that we enroll may introduce a selection bias and elder FEVR children should be observed in future. Likewise, the follow-up time ought to be extended. Third, due to the X-linked recessive manner of NDP mutation, there may exist potential bias in statistical analysis. Finally, cultural or social factors may affect family decision making, which may lead to unpredictable bias.

In conclusion, our long-term study suggested a correlation between clinical phenotypes and gene mutations in FEVR patients. Overall, we found LRP5 mutation accounted for the highest proportion. And the NDP mutation showed the severest clinical manifestation and KIF11 spontaneous mutation accounted for the majority. From the present evidence, we summarized that the main surgical methods for cases at Stage 4 to 5 were transcorneal vitrectomy with lensectomy or LSV and the proportion of surgical treatment was much higher in patients under 1 year old, indicating that the earlier FEVR occurred, the worse the prognosis would be. Consequently, active surgical intervention and lens sparing were necessary for the cases at Stage 4 to 5.

## Data Availability

The datasets used and/or analyzed during the current study are available from the author P.Z on reasonable request.

## Abbreviations

FEVR: Familial exudative vitreoretinopathy
Closed-V: Closed vitrectomy
PPV: Pars plana vitrectomy
RD: Retinal detachment
LSV: Lens-sparing vitrectomy
SB: Scleral buckling
IVR: Intravitreal injection of ranibizumab
SPSS: Statistical Product and Service Solutions
MI: Monocular involvement
BM: Binocular involvement and mild manifestation
BS: Binocular involvement and severe manifestation
B(M + S): Binocular involvement and mild manifestation in one eye and severe manifestation in the other eye
MLCRD: Microcephaly-lymphedema-chorioretinal dysplasia
FFA: Fundus fluorescein angiography
VH: Vitreous hemorrhage
